# *Wolbachia* inhibits ovarian formation and increases blood feeding rate in female *Aedes aegypti*

**DOI:** 10.1371/journal.pntd.0010913

**Published:** 2022-11-11

**Authors:** Meng-Jia Lau, Perran A. Ross, Nancy M. Endersby-Harshman, Qiong Yang, Ary A. Hoffmann

**Affiliations:** Pest and Environmental Adaptation Research Group, Bio21 Institute and the School of BioSciences, The University of Melbourne, Parkville, Victoria, Australia; Fort Collins, UNITED STATES

## Abstract

*Wolbachia*, a gram-negative endosymbiotic bacterium widespread in arthropods, is well-known for changing the reproduction of its host in ways that increase its rate of spread, but there are also costs to hosts that can reduce this. Here we investigated a novel reproductive alteration of *Wolbachia w*AlbB on its host *Aedes aegypti*, using studies on mosquito life history traits, ovarian dissection, as well as gene expression assays. We found that an extended period of the larval stage as well as the egg stage (as previously shown) can increase the proportion of *Wolbachia*-infected females that become infertile; an effect which was not observed in uninfected females. Infertile females had incomplete ovarian formation and also showed a higher frequency of blood feeding following a prior blood meal, indicating that they do not enter a complete gonotrophic cycle. Treatments leading to infertility also decreased the expression of genes related to reproduction, especially the vitellogenin receptor gene whose product regulates the uptake of vitellogenin (Vg) into ovaries. Our results demonstrate effects associated with the development of infertility in *w*AlbB-infected *Ae*. *aegypti* females with implications for *Wolbachia* releases. The results also have implications for the evolution of *Wolbachia* infections in novel hosts.

## Author summary

*Wolbachia* bacteria reduce the transmission of dengue and other arboviruses transmitted by their mosquito hosts once they have invaded host populations. This invasion process is being undertaken in *Aedes aegypti* mosquito populations throughout the world but can be slowed by deleterious fitness costs that the *Wolbachia* bacteria have on their mosquito hosts. Here we investigate in detail a recently identified cost where one of the *Wolbachia* strains being used in invasions causes infertility in females when the females develop from eggs that have been stored for a period. We show that slowed larval development (through food reduction) can cause the same phenomenon and that the emerged females lack functional ovaries. These infertile females also take blood meals more often. The results have implications for the successful invasion of *Wolbachia* under ecological circumstances where the mosquito eggs have to persist in the absence of water for a period and where larval food is limiting.

## Introduction

*Wolbachia*, a *Rickettsia*-like maternally inherited endosymbiotic bacterium widespread in insects [1], can alter insect reproduction in multiple ways to enhance its transmission [2]. Specific alterations include parthenogenesis–infected females can produce female offspring without mating; feminization–infected genetic males are transformed into functional females and produce female offspring; male killing–infected immature males die while females survive, and cytoplasmic incompatibility (CI)–females infected with the predominant *Wolbachia* strain obtain a fertility advantage because males infected with *Wolbachia* can cause female embryonic lethality when they mate with uninfected females or females infected with a different *Wolbachia* strain [3,4]. Aside from these alterations that enhance the spread of *Wolbachia*, there can also be costs to the infection that slow its spread [5–7]. These effects of *Wolbachia* are normally based on investigations of natural *Wolbachia* strains that have existed in their hosts for many years, but are also frequently found in artificial infections [8].

In the last decade, several artificially introduced *Wolbachia* strains have been successfully used to reduce the transmission of arboviral diseases by *Aedes aegypti* mosquitoes [9,10], including field releases with the *Wolbachia* strains *w*Mel [11–13] and *w*AlbB [14]. Studies on native hosts tend to indicate only a weak fitness cost of *Wolbachia* [6], but *Wolbachia* can have diverse fitness costs in its newly introduced host *Ae*. *aegypti*, such as life-shortening, heat sensitivity, reduced quiescent egg viability and reduced blood feeding success [10,15–17]. The effects of *Wolbachia* in native and introduced hosts point to the possibility of a mutualistic relationship between *Wolbachia* developing over time because of co-evolution and adaptation. In fact, it is acknowledged that sometimes *Wolbachia* can act as both parasite and mutualist [5,18,19]. As a result, *Wolbachia* field release and establishment in new populations can be useful for studying evolutionary changes in *Wolbachia* given that the invasion history of the population is known [20–22].

In our previous study of the effects of *Wolbachia w*Mel and *w*AlbB on *Ae*. *aegypti* eggs, we discovered a novel reproductive effect induced by *w*AlbB, in which infected females that hatched from quiescent eggs were infertile despite successful mating and the proportion of females showing infertility was correlated with the duration of egg quiescence [23]. The *w*Mel strain also causes female infertility following egg storage, but to a lesser extent than *w*AlbB [23,24]. Several other reproductive fitness costs of *Wolbachia*, including those on the number and viability of eggs, can be attributed to the consequence of nutritional competition between *Wolbachia* and its host [25] and represent quantitative effects. However, in our study *Wolbachia*-infected females lost their fertility entirely, indicating a qualitative effect difficult to attribute to nutritional competition [23], so we suspect the involvement of other mechanisms.

In this paper, we investigate factors associated with *Ae*. *aegypti* female infertility induced by *Wolbachia w*AlbB infection after egg quiescence. Firstly, we tested if the effects of *Wolbachia* on female infertility are permanent and associated with a lack of fully developed ovaries. Secondly, we performed a larval starvation experiment to see if an extended larval period might also affect infertility. Thirdly, we undertook real-time PCR assays to understand the interaction between *Wolbachia* and the expression of genes influencing female development. Finally, we tested whether the loss of fully developed ovaries in infertile females affected the rate of female blood feeding. Our study highlights the novel nature of reproductive effects altered by *Wolbachia w*AlbB in its new host *Ae*. *aegypti*, with implications for invasion of *Wolbachia* into uninfected populations and the evolution of mutualism more generally.

## Methods

### Ethics statement

The process of mosquito females feeding on human volunteers is approved by the University of Melbourne Human Ethics committee (approval 0723847). All adult subjects provided informed written consent (no children were involved).

### Mosquito populations

We used uninfected and *Wolbachia w*AlbB-infected *Aedes aegypti*. The uninfected population was derived from eggs collected in 2019 from regions in Cairns, Queensland, Australia where *Ae*. *aegypti* were not infected with *Wolbachia*. The *w*AlbB-infected population was generated by microinjection [26] and infected females were crossed to males from the uninfected population regularly [27,28] to maintain a similar genetic background between populations. Mosquitoes were maintained in the laboratory following methods described previously [29] and all mosquito populations were screened routinely for their infection status. In the following experiments, non-stored *w*AlbB-infected and uninfected females were hatched from freshly-laid eggs that had been dried and conditioned for one week at 26 ± 1°C, and stored *w*AlbB-infected females were hatched from eggs that had been stored for two to four months at 26 ± 1°C. All lines tested in the same experiment had the same rearing and feeding conditions. In all experiments, female mosquitoes were blood fed on the same volunteer, and in each comparison, blood feeding of all cages was finished within two hours.

### Determination of female fertility status

To determine the fertility status of individual females in the experiments, we hatched a mixture (approximately 1:1) of *w*AlbB-infected eggs that had been stored for two and four months, so that the proportion of infertile females is expected to be 25% and 80% respectively [23]. Female mosquitoes were blood fed on 3–5 days post-emergence. 200 engorged females were aspirated individually in 70 mL specimen cups with larval rearing water and sandpaper strips to allow them to lay eggs. After a week, we separated females that laid (fertile) or did not lay (infertile) eggs and grouped them separately in a *19*.*7-L BugDorm-1 adult cage (MegaView Science Co*., *Ltd*., *Taichung City*, *Xitun District*, *Taiwan)*. This process is referred to as “fertility separation”.

After “fertility separation”, we measured the relative *Wolbachia* density of fertile and infertile females for groups of 16 individuals based on the 2^ΔCt^ method [30]. Screening based on real-time PCR assays followed methods described previously [31]. For each replicate group, samples were set up in a 384-well white plate and density measured by primers *aeg* and *wMwA* [32]; two consistent replicates (ΔCt<1) were obtained per group and their values were averaged for the density determination.

To test whether females scored as infertile based on the above criteria recovered fertility later in their lives or remained infertile, we then provided both fertile and infertile females with a second blood meal after the “fertility separation” process mentioned in the last paragraph, and isolated 20 “fertile” and 20 “infertile” females individually in 70 mL cups for another week to observe if their fertility status changed. We dissected the remaining females scored as “fertile” and “infertile” to examine the appearance of their ovaries. These females were killed by storing them at -80°C for 30 minutes then returned to room temperature for 10 minutes before dissecting them in saline solution (0.9% sodium chloride) under a compound light microscope (Motic B1 series, Australian Instrument Services Pty. Ltd., Australia).

To further examine fertility effects, we also dissected and examined ovaries in 65 *w*AlbB-infected females that had been stored as eggs for 11 weeks and that were 3–5 days post-emergence, but without a blood meal. These females had not been exposed to the “fertility separation” process but should show a high rate of infertility [23]. We used an NIS Elements BR imaging microscope (Nikon Instruments, Japan) to photograph the ovary tissues. The same dissections were also undertaken for a *w*AlbB transinfection line that had been repeatedly backcrossed to a Saudi Arabian background [33]. In this case we dissected ovaries from 113 *w*AlbB-infected mosquitoes with an egg stage that had lasted for 12 weeks, as well as 50 *w*AlbB-infected females with an egg stage that had lasted for one week and 50 uninfected Saudi Arabian females with an egg stage that had lasted for 12 weeks (S1 Table).

### Impact of larval starvation on mosquito infertility

To investigate whether the impact of *Wolbachia* infection on female infertility was only mediated through the egg stage or whether the prepupal stage had an impact as a whole, we deprived 2^nd^ instar mosquito larvae of food for two weeks before feeding them again until pupation. We also set up non-starved controls where larvae were provided with food *ad libitum*. Each starvation treatment was performed with both stored eggs (egg stage had lasted for 12 weeks) and non-stored eggs (egg stage had lasted for one week) for a total of four treatments to test if any fertility effects might accumulate across the larval and egg stages. In each treatment, we hatched 400 larvae and reared them in a tray containing 4 L of reverse osmosis water [29]. Before the blood meal, 15 individual females randomly selected from each treatment (3–5 days post-emergence) were screened for *Wolbachia* infection and *Wolbachia* density, using the screening methods described in the previous section. To test the proportion of females that were infertile, we set up replicates consisting of groups of 30 individual females emerging from the same rearing tray. For the *w*AlbB-infected line, we had two replicates for the treatment in which infected females were neither stored nor starved, and three replicates for the other three experimental treatments. Female infertility was tested through the “fertility separation” process. For the control uninfected *Ae*. *aegypti* line, we set up all four similar treatments and dissected 30 individuals at 3–5 days post-emergence from each treatment to check for the presence of ovaries (S1 Table).

### Gene expression assays

We selected three genes related to mosquito reproduction and tested their expression levels in females at different developmental stages through a real-time PCR assay [31,32]. One of the genes tested was the *vitellogenin receptor* (*vgr*): following blood feeding, vitellogenin is secreted from the fat body and internalized by ovaries through the receptor VgR [34–36]. The other two genes were the *ecdysone receptor* (*ecr*) [37,38] and the *eggshell organizing factor* (*eof*); the vitellogenin transcript is regulated by *ecr* while *eof* is an essential gene encoding a protein for eggshell formation at the late stage of egg production [39] (S2 Table). Gene expression level was quantified relative to a control gene, *RPS17* [40]. We tested female pupae 0.5–1.5 days post-pupation and female adults 0.5–1.5 days post-emergence in *w*AlbB-infected females that hatched from stored eggs (egg stage had lasted for 14 weeks) and non-stored eggs (egg stage had lasted for one week) as well as non-stored uninfected females (egg stage had lasted for one week) considered as the control. For the *w*AlbB-infected females that had been stored as eggs, we also tested gene expression levels in fertile and infertile females after testing female fertility, and in this comparison fertile females were treated as the control. Mosquito samples were given a second blood meal and stored in RNA*later* (Sigma Aldrich Cat No. R0901-100ML) on the third day after feeding, before RNA extraction, reverse transcription and real-time PCR assays were conducted. In all of the above comparisons, we screened 10 individuals from each group; the details of RNA extraction and real-time PCR can be found in S1 Text.

### Blood feeding rate of female mosquitoes

Normally, fully engorged fertile females are reluctant to feed within a gonotrophic cycle. In this experiment, we tested egg storage impacts on subsequent feeding by females, sourcing mosquitoes from the same groups as used for the above gene expression assays. After determining female fertility, we measured the blood feeding rate of female mosquitoes by providing fertile and infertile females a third blood meal, three days after their second blood feeding, when engorged blood was almost digested. The same volunteer who provided previous blood meals fed the mosquitoes in a BugDorm cage for 15 minutes. Fully engorged females with blood visible in the abdomen were considered as having successfully fed after this feeding attempt. Three replicates were completed with 20–30 individual females for each replicate. We also weighed 20 random females that had fully fed and 20 that had not been provided with a blood meal from all three replicates to estimate their blood meal weight using a Sartorius Analytical balance BP 210 D (Sartorius, Gottigen, Germany, Readability: 0.01 mg).

### Statistics

We used R v. 3.6.0 with R studio v. 1.1.453 to conduct data analyses and visualizations [41], using the “car” library for ANOVA [42], the base library for other statistical analyses, and the “ggplot2” library for visualization [43]. After real-time PCR measurement, we used the 2^–ΔΔCt^ method to compare gene expression levels [44], the values of 2^–ΔΔCt^ were natural log-transformed for ANOVA analysis, and a Tukey’s honest significant difference (HSD) test [45] was used for further pairwise comparisons. For *Wolbachia* density we used the 2^ΔCt^ method [30] to calculate relatively density before log10 transformation for ANOVA analysis. For the proportion data from larval starvation and blood feeding experiments, we analysed data with binomial logistic regression models [46]. In the blood feeding rate test, student’s t-tests were used to test for changes of mosquito weight after blood feeding. Treatments in this study are listed in S3 Table.

## Results

### Immature ovaries in infertile *Aedes aegypti* females

**Fig 1:**
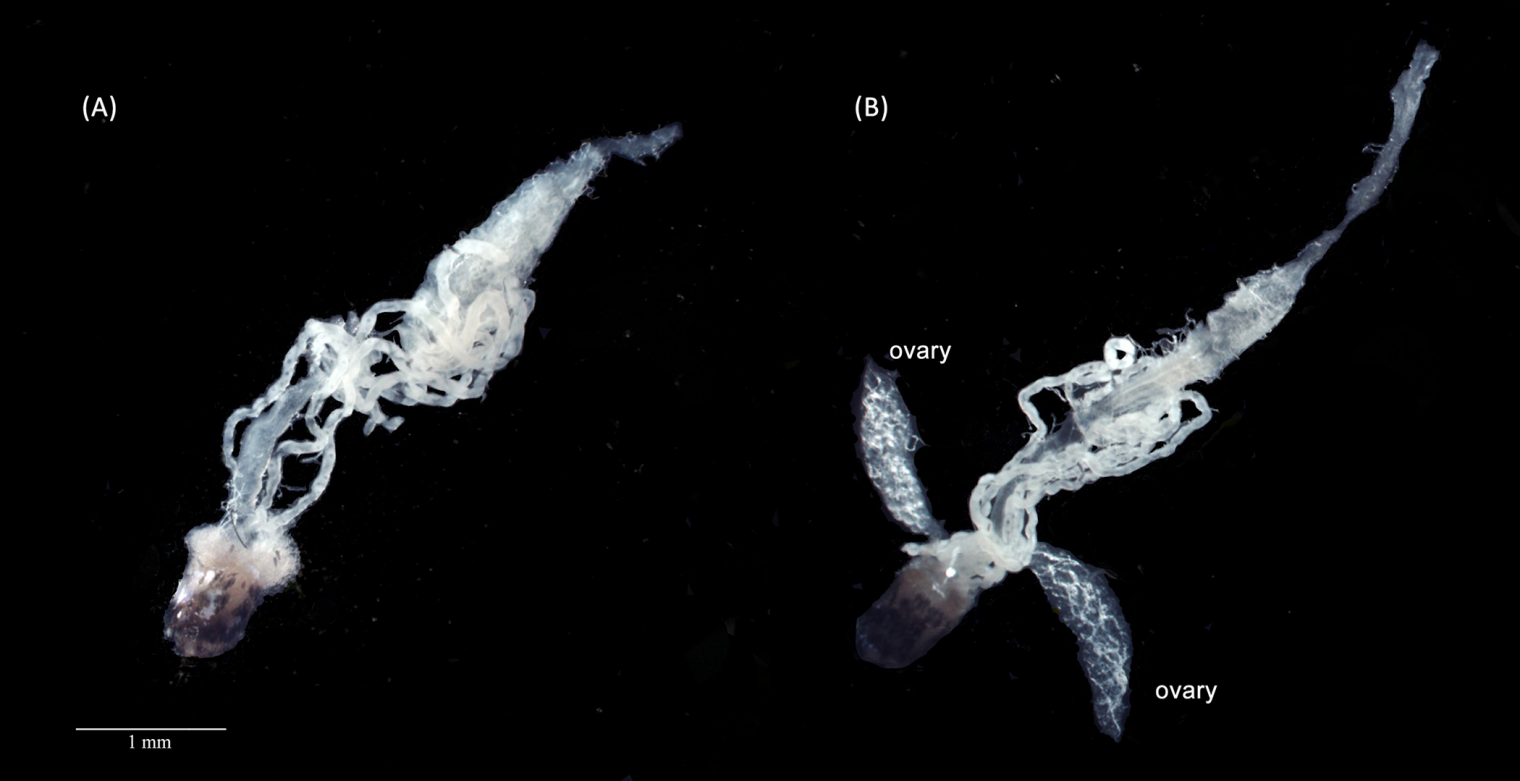
Appearance of internal organs in *w*AlbB-infected *Aedes aegypti* mosquitoes. (A) Infertile females lack observable ovaries, while ovaries are present in (B) fertile females (labelled as “ovary”). The background of the photographs was removed for clarity, with the original photographs presented in S3 Fig.

We performed a “fertility separation” for *w*AlbB-infected females and provided a second blood meal to fertile and infertile females. We confirmed that all 20 females we scored as fertile and the 20 we scored as infertile after the first gonotrophic cycle maintained their phenotype in the second gonotrophic cycle. We dissected and compared the ovarian appearance of fertile and infertile females and discovered that developed ovaries could not be observed in infertile females under a microscope (Fig 1). Only occasionally (around one in ten) can immature ovarian structure be seen, but with a narrow width similar to that of a Malpighian tube (S1 Fig). We also found that fertile females had higher relative densities of *Wolbachia* (S2 Fig: ANOVA comparing fertile and infertile females: F_1,30_ = 8.632, P = 0.006, fertile: mean ± se = 5.15 ± 0.906; infertile: mean ± se = 2.290 ± 0.673).

For the 3–5 days post-emergence mosquitoes dissected without separating them into fertile and infertile categories, we found that the majority of *w*AlbB females stored as eggs for 11–12 weeks lacked visible ovaries (S1 Table) in contrast to the uninfected population and females that did not emerge from stored eggs. This pattern was also evident in the Saudi Arabian populations which had a different genetic background (S1 Table).

### Impact of larval starvation on mosquito infertility

**Fig 2:**
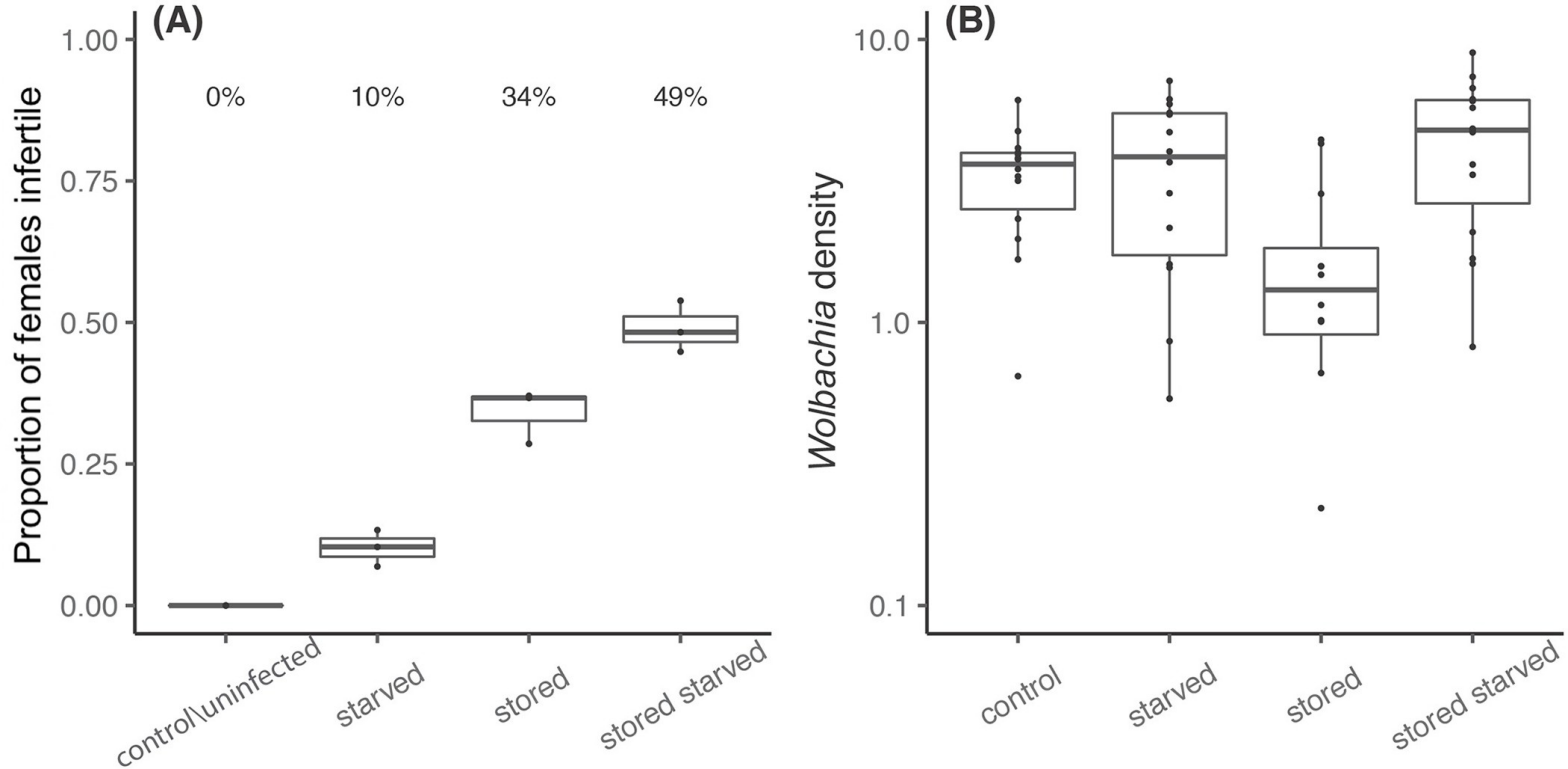
(A) Boxplots showing the proportion of infertile females in *w*AlbB-infected and uninfected mosquitoes with an egg stage that had lasted for 12 weeks (stored) or one week and a larvae stage that had been deprived of food for two weeks (starved) or that had not been deprived of food. Mosquitoes that had not been treated with "stored" or “starved” are designated as “control”. No uninfected females from all four treatments were infertile. Values above boxplots represent corresponding averaged percentages based on two replicates for controls and three replicates for treatments with each replicate containing 30 individuals. (B) Boxplots of relative *Wolbachia* density in 3–5 days post-emergence females with unknown fertility status. Each group is based on two consistent real-time PCR replicates.

We deprived mosquito larvae of food to increase the duration of the larval stage from approximately one week to three weeks to test for any impact of larval starvation on infertility in *w*AlbB-infected females. We found significant effects of both storage and starvation treatment, with an interaction between these terms (Fig 2A: logistic regression testing effects of storage: F_1,7_ = 256.304, p < 0.001; starvation: F_1,7_ = 32.412, p < 0.001; interaction: F_1,7_ = 21.515, p = 0.002). Infertility associated with *w*AlbB infection increased further after larval starvation over and above that seen with stored eggs by 15%, and the extension of the larval stage by a period of starvation also induced infertility of around 10% by itself. For uninfected *Ae*. *aegypti* treated in the same manner, all females that were dissected contained ovaries, indicating the essential role of *Wolbachia w*AlbB in inducing female infertility during larval starvation and egg storage (Fig 2A and S1 Table).

Before blood feeding to identify female fertility status, we collected 15 females of 3–5 days post-emergence to screen for their *Wolbachia* infection status. We failed to detect an infection in five out of 60 individuals and these were excluded from the density analysis (S4 Table). *Wolbachia* density was significantly influenced by larval starvation but not egg quiescence, with a significant interaction (Fig 2B: ANOVA: starvation: F_1,51_ = 7.145, p = 0.010; egg quiescence: F_1,51_ = 2.475, p = 0.122, interaction between starvation and quiescence: F_1,51_ = 8.396, p = 0.006). It is likely that egg quiescence decreased *Wolbachia* density, but density increased with larval starvation, potentially cancelling out this effect [47].

### Gene expression assays

**Fig 3:**
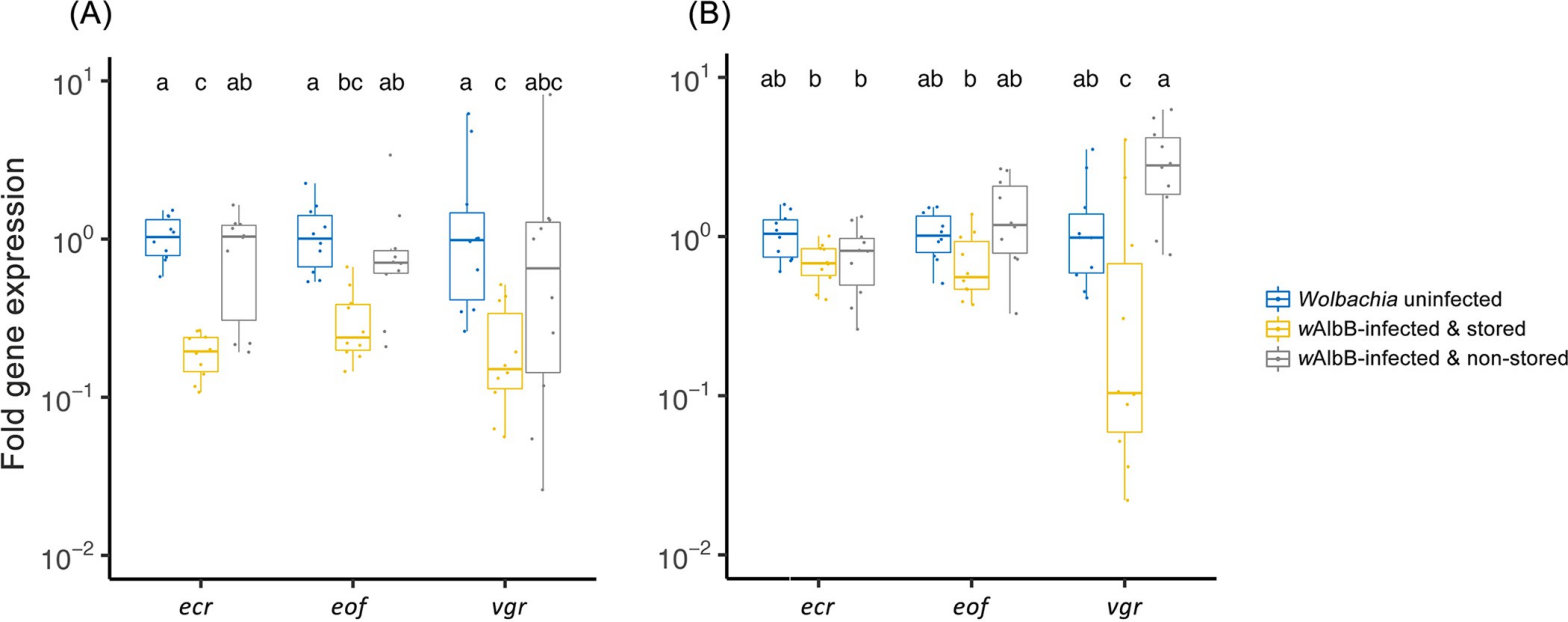
Boxplots of relative expression of reproduction-related genes *ecdysone receptor* (*ecr*), *eggshell organizing factor* (*eof*) and *vitellogenin receptor* (*vgr*) in female (A) pupae and (B) young adults (1 ± 0.5 days post-emergence). These three genes were normalized to reference gene *RPS17*. Values with the same letter are not significantly different based on Tukey’s HSD tests (S5 Table). Results are based on two consistent real-time PCR replicates of ten individual females from each group.

We selected three genes related to reproductive development and tested their expression level at different developmental stages. At the pupal stage, there were significant differences on gene expression among the three mosquito lines (*w*AlbB-infected with an egg stage that had lasted for 14 weeks; *w*AlbB-infected and *Wolbachia* uninfected with an egg stage that had lasted for only one week (non-stored)), but no interaction between lines and the genes or overall difference between the genes (Fig 3A, ANOVA on relative expression: mosquito group: F_2,81_ = 26.717, p < 0.001; genes: F_2,81_ = 1.044, p = 0.357; interaction: F_4,81_ = 0.423, p = 0.792). Specifically, in Tukey’s HSD tests, *w*AlbB-infected females hatched from stored eggs had lower expression levels for all three genes than uninfected females, while only *ecr* had a lower expression level in *w*AlbB-infected females that had been stored compared to infected females that not been stored (Fig 3A).

For young adults 0.5–1.5 days post-emergence, significant differences in gene expression levels were found among mosquito groups, but there was no interaction with the genes (Fig 3B, ANOVA on relative gene expression: mosquito group: F_2,81_ = 15.641, p < 0.001; genes: F_2,81_ = 0.383, p = 0.683; interaction: F_4,81_ = 7.100, p = 0.792). Specifically, only differences for *vgr* expression were significant between *w*AlbB-infected females that been stored and the other two mosquito groups, with some individuals from stored eggs having very low expression levels—these females are probably infertile (Fig 3B).

**Fig 4:**
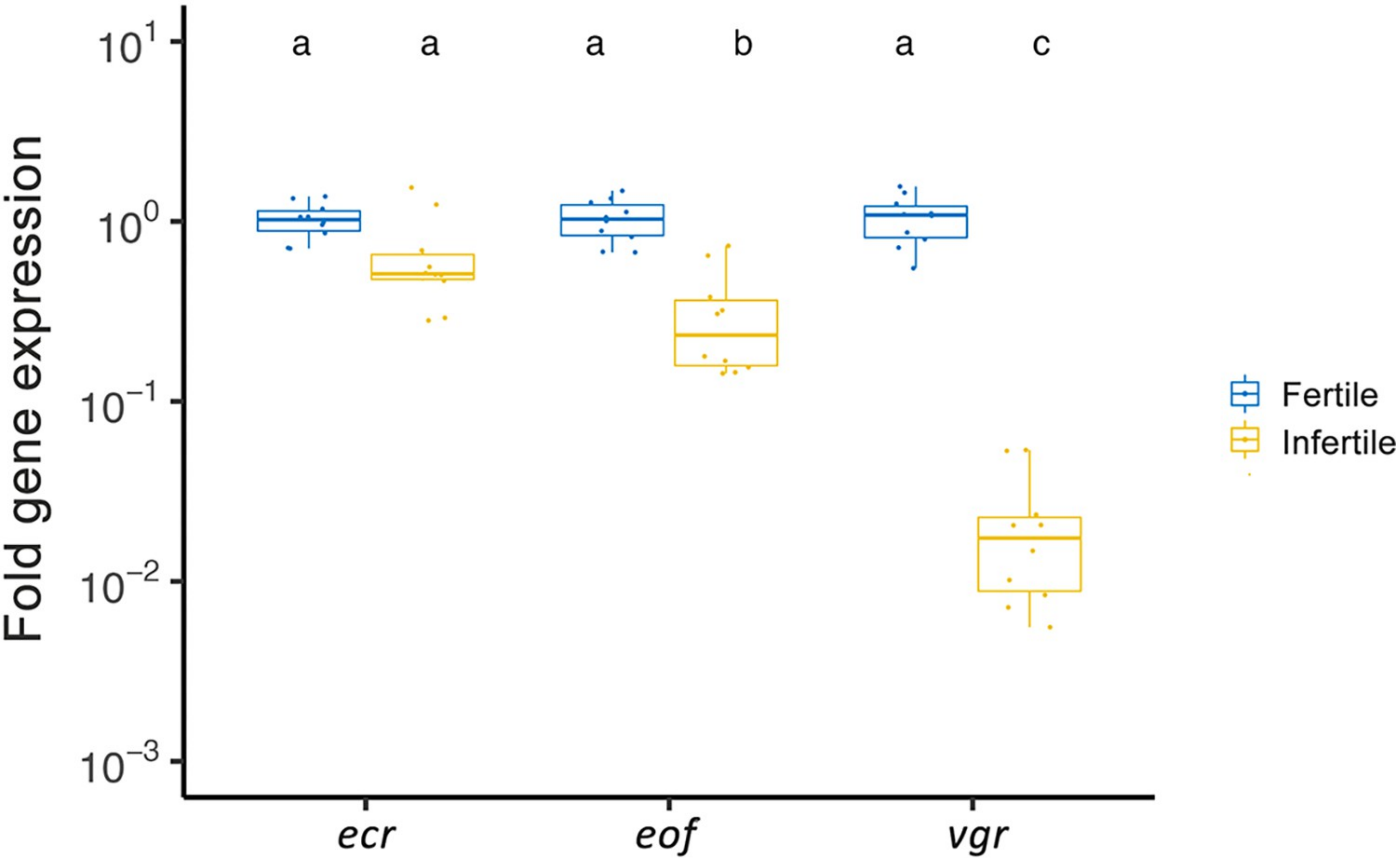
Boxplots of relative expression of reproduction-related genes *ecdysone receptor* (*ecr*), *eggshell organizing factor* (*eof*) and *vitellogenin receptor* (*vgr*) in fertile and infertile females one week after their second blood meal. Genes were normalized to reference gene *RPS17*. Values with the same letter are not significantly different according to Tukey’s HSD tests. Results are based on two consistent real-time PCR replicates of ten individual females from each group.

After “fertility separation”, female mosquitoes were provided a second blood meal and were screened for gene expression levels three days later. Significant differences were found between fertile and infertile females, and also between genes and their interaction (Fig 4, ANOVA on relative gene expression: mosquito group: F_1,54_ = 235.696, p < 0.001; genes: F_2,54_ = 68.553, p < 0.001; interaction: F_2,54_ = 68.553, p < 0.001). Genes *eof* and *vgr* showed significant differences between fertile and infertile females for expression, especially in the case of *vgr* whose expression in infertile mosquitoes was only around 0.01% of that seen in fertile mosquitoes (Fig 4), likely reflecting the fact that infertile females had incomplete ovarian formation.

### Blood feeding rate

**Fig 5:**
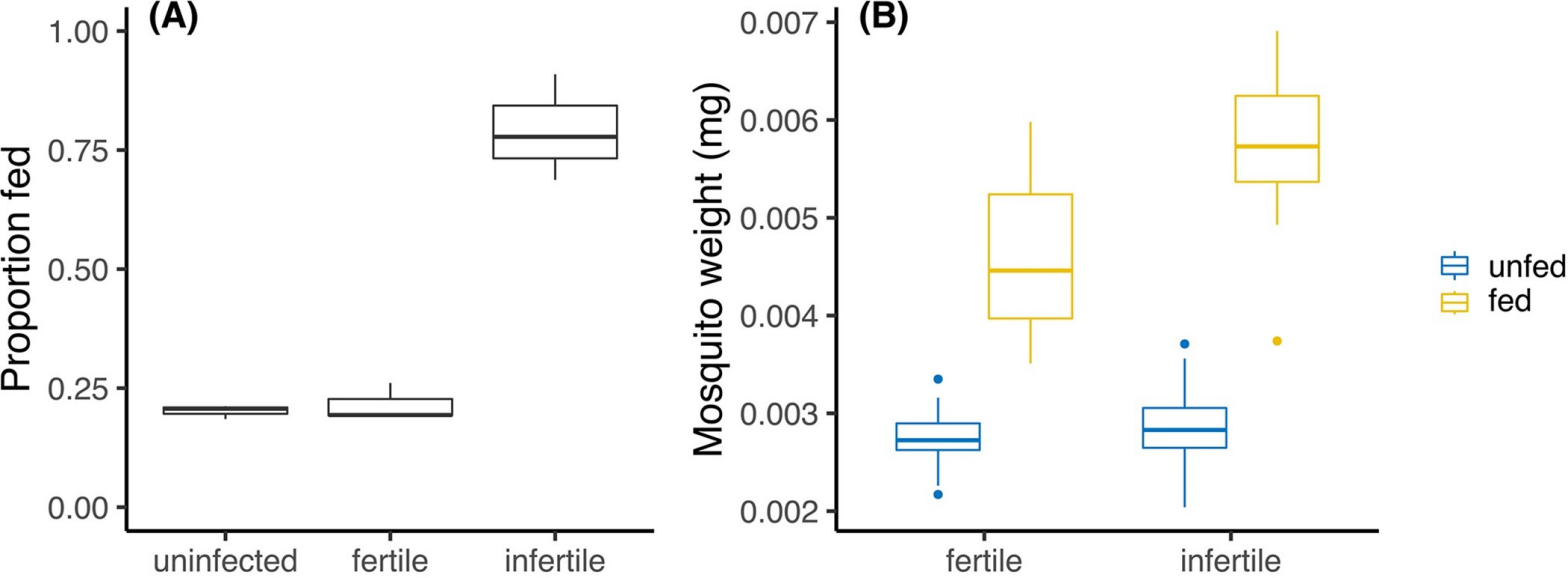
Boxplots of (A) female proportion that blood fed three days after they had been fully fed, in which three replicates were completed with 20–30 individual females for each replicate; and (B) the weights of fed and unfed females. 20 females that had fully fed and 20 that had not been provided with a blood meal from all three replicates were weighed at random.

After “fertility separation”, we provided female mosquitoes a third blood meal on the third day after they had become engorged with their second blood meal to test their blood feeding behaviour. Infertile females had a higher proportion feeding compared to fertile and uninfected females (Fig 5A, logistic regression, F_2,6_ = 61.037, p < 0.001). We also compared the weight of fully fed and unfed females and noted significant differences between mosquito groups, feeding status and their interaction (Fig 5B, ANOVA: mosquito group: F_1,73_ = 29.06, p < 0.001; feeding: F_1,73_ = 322.89, p < 0.001; interaction: F_2,73_ = 15.26, p < 0.001). Specifically, significant weight increases were recorded for both *w*AlbB-infected fertile females (t_35_ = 9.627, p < 0.001) and *w*AlbB-infected infertile females (t_38_ = 15.658, p < 0.001), but the weight of fully fed fertile females was significantly lower compared to infertile females (t_35_ = -4.645, p < 0.001), while there was no significant difference between unfed fertile and infertile females (t_38_ = -1.096, p = 0.280), suggesting that infertile females took in a larger amount of blood during successive feeding periods.

## Discussion

*Wolbachia* is a well-known bacterium that can alter the reproduction of its host to benefit its spread. In our previous study, *Wolbachia w*AlbB was found to cause *Ae*. *aegypti* females to become infertile following egg storage [23], inhibiting vertical transmission. In this paper, we investigated this phenomenon and obtained the following results: 1) in infertile females, the development of ovaries has been interrupted by *Wolbachia*, which is supported by ovarian dissection and gene expression assays; 2) the effect of *Wolbachia* on female fertility accumulates across pre-pupal stages, in that the frequency of female infertility depends on the duration before metamorphosis (including egg and larval stages); and 3) infertile females maintain other female characteristics such as mating and blood feeding, but they do not enter a gonotrophic cycle and blood feeding occurs more frequently. We, therefore, confirmed a novel reproductive alteration of *Wolbachia w*AlbB in its new host, *Ae*. *aegypti* when coupled with an extended pre-pupal stage.

The effect of *Wolbachia* on female infertility is different from other fitness impacts of *Wolbachia* and the four typical reproductive alterations (parthenogenesis, feminization, male killing and cytoplasmic incompatibility) in which *Wolbachia* alters host reproduction in ways that increase its vertical transmission success [4,48]. The infertility effect we describe here converts functional females into non-functional females without male characteristics, suggesting different *Wolbachia*-related mechanisms to those involved in other phenomena like feminization [49,50]. By testing the expression level of *vgr*, *ecr* and *eof* at the female pupal, and adult stages before and after blood feeding, we found substantial differences in gene expression between fertile and infertile groups. For pupae, large variation in expression levels were found among individuals, especially for expression of the gene *vgr*, probably reflecting the fact that its expression mainly occurs in a narrow period during the pupal stage. *w*AlbB-infected *Ae*. *aegypti* females that had been stored long-term as eggs had lower expression levels of the three reproductive genes compared with uninfected females, especially for the gene *vgr* at the young adult stage, suggesting the formation and development of oocytes was impacted. This is also supported by the very low relative expression of *vgr* and *eof* in infertile females when compared with fertile females at the third day after blood feeding, and the absence of mature ovaries in infertile females. The lower overall *Wolbachia* densities in infertile females is also consistent with previous work showing high *w*AlbB densities in ovaries (S2 Fig) [51]. Moreover, we found much lower expression of *ecr* in *w*AlbB-infected female pupae hatched from long-stored eggs, while this difference was not found in young adults. As *ecr* is expressed in a variety of organs to encode the receptor of a hormone [52], the down-regulation of *ecr* may indicate that egg quiescence has a substantial impact on the pupation process of *w*AlbB-infected females. However, interactions between *Wolbachia* and specific reproductive signals and mechanisms are poorly understood and require further investigation for example, through hormone level tests, transcriptome analysis and pathway analysis [53,54].

In population replacement approaches [8], the novel reproductive alteration induced by *Wolbachia w*AlbB could slow the spread of the infection in mosquito populations as females are less likely to produce offspring. This may occur not only when the egg desiccation period is extended during dry and warm seasons [55,56], but also when the larval period is extended due to poor provision of food [57–59]. These effects can reduce the efficiency of *Wolbachia* invasion which aims to inhibit the transmission of arboviruses [8,28,60]. While *w*AlbB has established successfully in Malaysia where the warm and humid year-round climate precludes extended egg desiccation periods and promotes fast development of larvae [14,61], our discovery provides guidance for future releases in other climates and highlights the importance of future monitoring [23]. Our results also have implications for laboratory studies and release programs where it is common to store large numbers of eggs prior to experiments and transportation to release sites.

Our observation that infertile females show an increased rate of blood feeding highlights a potential risk of increased nuisance biting following a *Wolbachia* release program, which may lead to community discontentment. Normal *Ae*. *aegypti* females ingest human blood to obtain unique nutrients for egg development [62,63]. However, while infertile females lack ovaries, they still blood feed. Previous research found that multiple feeding within a gonotrophic cycle is caused by nutritional reserve depletion or feeding interruption [64]. Nevertheless, we found a large proportion of infertile females fed again on the third day after their second blood meal, indicating they do not follow the same gonotrophic cycle. It is unclear if and how the process of blood digestion is impacted by infertility. Infertile females also took in a larger amount of blood, though this might be a compensatory reaction towards starvation at egg and larval stages. This behavioural change of *Wolbachia*-infected infertile females has significant implications for vector control population release strategies [8,65]; in particular it would be interesting to test whether the ability of these infertile females to carry and transmit arbovirus has changed as the density of their *Wolbachia* infection may be lower than that of fertile females [66,67]. Similar concerns have been raised about increased biting frequencies of irradiated female mosquitoes in sterile insect technique (SIT) programs [68], however in population replacement approaches the prevalence of infertile females is likely to be much higher in *Wolbachia-*infected mosquito populations compared to in SIT irradiated females that are only released accidentally.

The reproductive alterations of *Wolbachia* discovered in this study have evolutionary implications. So far, our work relates to *Ae*. *aegypti*, where the *Wolbachia w*AlbB was artificially introduced recently from a close relative of *Ae*. *aegypti*, *Aedes albopictus*, in order to reduce the transmission of arboviral diseases [69]. A *Wolbachia* strain may induce similar fitness costs in a new host when compared to its native host, such as in the case of life-shortening caused by *w*MelPop which is expressed in both *Drosophila melanogaster* and *Ae*. *aegypti* [10], and remains stably expressed following long-term laboratory culture [70]. We do not yet know if there are similar reproductive effects of *Wolbachia w*AlbB expressed in its native host, *Ae*. *albopictus*. On the other hand, we have also discovered a smaller loss of female infertility in *w*Mel infected *Ae*. *aegypti* [23], though the native host of *w*Mel, *D*. *melanogaster*, does not enter egg quiescence, although there is a reduced fecundity of *w*Mel-infected females under dormancy conditions [71]. Although other *Wolbachia* strains remain to be investigated, it is possible that *Ae*. *aegypti* has mechanisms underlying ovarian formation that can be interrupted by endosymbionts. However, it is unclear why female infertility is observed in *Wolbachia*-infected mosquitoes after increased periods of egg storage or larval development time, and to what extent the duration of pre-pupal stage that causes female infertile will be impacted by mosquito genetic backgrounds. There may be cumulative effects of *Wolbachia* at early stages of development contributing to failure of ovarian formation. One possible hypothesis which requires further investigation is that *Wolbachia* competes for some essential but sparse nutrients with its host *Ae*. *aegypti* at an early life stage, such as amino acids and cholesterol that mainly come from human blood [72,73].

In conclusion, we further investigated a novel reproductive alteration of *Wolbachia* that we discovered previously [23]. We confirmed that *Ae*. *aegypti* females infected with *Wolbachia w*AlbB can become infertile when they are unable to form functional ovaries during metamorphosis, but these females retain other female characteristics, leading to an increased biting frequency. Our study provides significant guidance for future *Wolbachia* releases and has important evolutionary implications for understanding the reproductive alteration of *Wolbachia*, especially in novel hosts.

## Supporting information

S1 Table Summary of the number of mosquitoes dissected without “fertility separation” and their ovarian developmental status. (DOCX)

S2 Table Primers used to measure the expression of three essential reproductive-related genes in *Aedes aegypti* females. (DOCX)

S3 Table List of treatments and their definitions. (DOCX)

S4 Table Summary of the number of uninfected mosquitoes that were excluded in the density analysis in the *Aedes aegypti* larval starvation experiment. (DOCX)

S5 Table Density means and results of posthoc tests distinguishing the groups (different letters indicate significant differences between the means) based on data presented in Figs 3 and 4. (DOCX)

S1 Text Methods for expression analysis. (DOCX)

S1 Fig An uncommon case where immature ovarian structures can be seen in infertile females, with the width of ovaries similar to Malpighian tubules. (DOCX)

S2 Fig Box plots of relative *Wolbachia* density of fertile and infertile *w*AlbB-infected females after females were separated into fertile and infertile groups (one week after blood meal). (DOCX)

S3 Fig Original pictures for Fig 1. (DOCX)
