# Supplementary material for: Wolbachia inhibits ovarian formation and increases blood feeding rate in female Aedes aegypti
Source: PLoS Negl Trop Dis. 2022 Nov 11;16(11):e0010913. doi: 10.1371/journal.pntd.0010913 (PMC9683608; doi:10.1371/journal.pntd.0010913)
Supplement: S1 Table — (DOCX) [file pntd.0010913.s001.docx]

**S1 Table.** Summary of the number of mosquitoes dissected without “fertility separation” and their ovarian developmental status.

| Mosquito genetic background | *Wolbachia* infection | Egg storage duration | Larval starvation | Number with ovaries present | Number with ovaries absent | Number with ovaries immature |
| --- | --- | --- | --- | --- | --- | --- |
| Australia | *w*AlbB | 11 weeks | No | 29 | 33 | 3 |
| Saudi Arabia | *w*AlbB | 12 weeks | No | 49 | 57 | 7 |
| Saudi Arabia | *w*AlbB | 1 week | No | 49 | 1 | 0 |
| Saudi Arabia | uninfected | 12 weeks | No | 50 | 0 | 0 |
| Australia | uninfected | 1 week | No | 30 | 0 | 0 |
| Australia | uninfected | 12 weeks | No | 30 | 0 | 0 |
| Australia | uninfected | 1 week | Yes | 30 | 0 | 0 |
| Australia | uninfected | 12 weeks | Yes | 30 | 0 | 0 |
