# Supplementary material for: Wolbachia inhibits ovarian formation and increases blood feeding rate in female Aedes aegypti
Source: PLoS Negl Trop Dis. 2022 Nov 11;16(11):e0010913. doi: 10.1371/journal.pntd.0010913 (PMC9683608; doi:10.1371/journal.pntd.0010913)
Supplement: S2 Table — (DOCX) [file pntd.0010913.s002.docx]

**S2 Table.** Primers used to measure the expression of three essential reproductive-related genes in *Aedes aegypti* females.

| Primer name* | Targeted Open reading frame (ORF) | Sequence (5'->3') | Amplicon size (bp) | Efficiency | Source |
| --- | --- | --- | --- | --- | --- |
| ecr838_F | Ecdysone receptor  AAEL009600 | CGGAAGGAGAAGAAAGCCCA | 80 | 83.77% | This study |
| ecr838_R |  | CGGTAGGTGCTGTTCGTTGT |  |  |  |
| eof696_F | eggshell organizing factor  AAEL012336 | TCCGACCTTGAGCAGCAAAT | 75 | 81.64% | This study |
| eof696_R |  | TTGCTTGCTGGGAGTCTGAG |  |  |  |
| vgr563_F | Vitellogenin receptor  AAEL014222 | GCTTCCGTCGGTACAATCCT | 93 | 86.46% | This study |
| vgr563_R |  | GCCTGTGCCGAGAATGAGTA |  |  |  |
| rps17_F | Ribosomal Protein S17  AAEL004175 | AAGAAGTGGCCATCATTCCA | 200 | 89.04% | [1] |
| rps17_R |  | GGTCTCCGGGTCGACTTC |  |  |  |

*Primer name end with ‘_F’ represents forward primer, end with ‘_R’ represents reverse primer, the two with the same prefix are a pair. Correlated genes are shown through the names of the primers.

[1] Dzaki N., Ramli K.N., Azlan A., Ishak I.H., Azzam G. 2017 Evaluation of reference genes at different developmental stages for quantitative real-time PCR in *Aedes aegypti*. *Sci Rep* **7**, 43618. (doi:10.1038/srep43618).
