# Supplementary material for: Wolbachia inhibits ovarian formation and increases blood feeding rate in female Aedes aegypti
Source: PLoS Negl Trop Dis. 2022 Nov 11;16(11):e0010913. doi: 10.1371/journal.pntd.0010913 (PMC9683608; doi:10.1371/journal.pntd.0010913)
Supplement: S3 Table — (DOCX) [file pntd.0010913.s003.docx]

**S3 Table.** List of treatments and their definitions

| Fig 2 | |
| --- | --- |
| control | *w*AlbB-infected *Aedes aegypti* females with an egg stage that had lasted for one week at 26 ± 1℃ and larval stage that had been provided with enough food *ad libitum*. |
| stored | *w*AlbB-infected *Aedes aegypti* females with an egg stage had lasted for 12 weeks at 26 ± 1℃ and larval stage that had been provided with enough food *ad libitum*. |
| starved | *w*AlbB-infected *Aedes aegypti* females with an egg stage had lasted for one week at 26 ± 1℃ and larval stage that had been deprived of food for two weeks. |
| stored starved | *w*AlbB-infected *Aedes aegypti* females with an egg stage had lasted for 12 weeks at 26 ± 1℃ and larval stage had been deprived of food for two weeks. |
| Fig 3 | |
| *Wolbachia* uninfected | uninfected females with an egg stage that had lasted for one week at 26 ± 1℃. |
| *w*AlbB-infected & stored | *w*AlbB-infected uninfected females with an egg stage that had lasted for 14 weeks at 26 ± 1℃. |
| *w*AlbB-infected & non-stored | *w*AlbB-infected females with an egg stage that had lasted for one week at 26 ± 1℃. |
| Fig 4 | |
| Fertile | *w*AlbB-infected females with an egg stage that had lasted for 14 weeks at 26 ± 1℃ and had produced eggs one week after their blood feeding. |
| Infertile | *w*AlbB-infected females with an egg stage that had lasted for 14 weeks at 26 ± 1℃ and had not produced any eggs one week after their blood feeding. |
| Fig 5 | |
| uninfected | uninfected females with an egg stage that had lasted for one week at 26 ± 1℃. |
| Fertile | *w*AlbB-infected females with an egg stage that had lasted for 14 weeks at 26 ± 1℃ and had produced eggs one week after their blood feeding. |
| Infertile | *w*AlbB-infected females with an egg stage that had lasted for 14 weeks at 26 ± 1℃ and had not produced any eggs one week after their blood feeding. |
| unfed | *w*AlbB-infected fertile or infertile females that did not blood feed again three days after they had been fully engorged with blood. |
| fed | *w*AlbB-infected fertile or infertile females that blood fed again three days after they had been fully engorged with blood. |
