# Supplementary material for: Wolbachia inhibits ovarian formation and increases blood feeding rate in female Aedes aegypti
Source: PLoS Negl Trop Dis. 2022 Nov 11;16(11):e0010913. doi: 10.1371/journal.pntd.0010913 (PMC9683608; doi:10.1371/journal.pntd.0010913)
Supplement: S4 Table — (DOCX) [file pntd.0010913.s004.docx]

**S4 Table.** Summary of the number of uninfected mosquitoes that were excluded in the density analysis in the *Aedes* *aegypti* larval starvation experiment.

| Colonies | Storage (Y/N) | Starvation (Y/N) | Number of uninfected |
| --- | --- | --- | --- |
| control | N | Y | 1 |
| stored | Y | N | 3 |
| starved | N | Y | 0 |
| stored starved | Y | Y | 1 |
