# Supplementary material for: Wolbachia inhibits ovarian formation and increases blood feeding rate in female Aedes aegypti
Source: PLoS Negl Trop Dis. 2022 Nov 11;16(11):e0010913. doi: 10.1371/journal.pntd.0010913 (PMC9683608; doi:10.1371/journal.pntd.0010913)
Supplement: S5 Table — (DOCX) [file pntd.0010913.s005.docx]

**S5 Table.** Density means and results of posthoc tests distinguishing the groups (different letters indicate significant differences between the means) based on data presented in Fig 3/Fig 4.

| Fig 3A | | | |
| --- | --- | --- | --- |
| genes | mosquito lines | ln(relative gene expression) | Groups as defined by posthoc test |
| *ecr* | uninfected | 7.56E-17 | a |
| *ecr* | wAlbB-infected & stored | -1.70E+00 | c |
| *ecr* | wAlbB-infected & non-stored | -3.71E-01 | ab |
| *eof* | uninfected | 9.30E-17 | a |
| *eof* | wAlbB-infected & stored | -1.27E+00 | bc |
| *eof* | wAlbB-infected & non-stored | -3.41E-01 | ab |
| *vgr* | uninfected | -2.53E-16 | a |
| *vgr* | wAlbB-infected & stored | -1.77E+00 | c |
| *vgr* | wAlbB-infected & non-stored | -8.09E-01 | abc |
| Fig 3B | | | |
| genes | mosquito lines | ln(relative density) | groups |
| *ecr* | uninfected | -2.14E-16 | ab |
| *ecr* | wAlbB-infected & stored | -4.08E-01 | b |
| *ecr* | wAlbB-infected & non-stored | -3.55E-01 | b |
| *eof* | uninfected | 4.68E-16 | ab |
| *eof* | wAlbB-infected & stored | -4.51E-01 | b |
| *eof* | wAlbB-infected & non-stored | 1.83E-01 | ab |
| *vgr* | uninfected | 1.28E-16 | ab |
| *vgr* | wAlbB-infected & stored | -1.61E+00 | c |
| *vgr* | wAlbB-infected & non-stored | 9.36E-01 | a |
| Fig 4 | | | |
| genes | mosquito lines | ln(relative density) | groups |
| *ecr* | fertile | 2.96E-16 | a |
| *ecr* | infertile | -5.59E-01 | a |
| *eof* | fertile | -2.46E-16 | a |
| *eof* | infertile | -1.33E+00 | b |
| *vgr* | fertile | 4.44E-17 | a |
| *vgr* | infertile | -4.11E+00 | c |
