# Supplementary material for: Wolbachia inhibits ovarian formation and increases blood feeding rate in female Aedes aegypti
Source: PLoS Negl Trop Dis. 2022 Nov 11;16(11):e0010913. doi: 10.1371/journal.pntd.0010913 (PMC9683608; doi:10.1371/journal.pntd.0010913)

**S1 Fig.** An uncommon case where immature ovarian structures can be seen in infertile females, with the width of ovaries similar to Malpighian tubules.


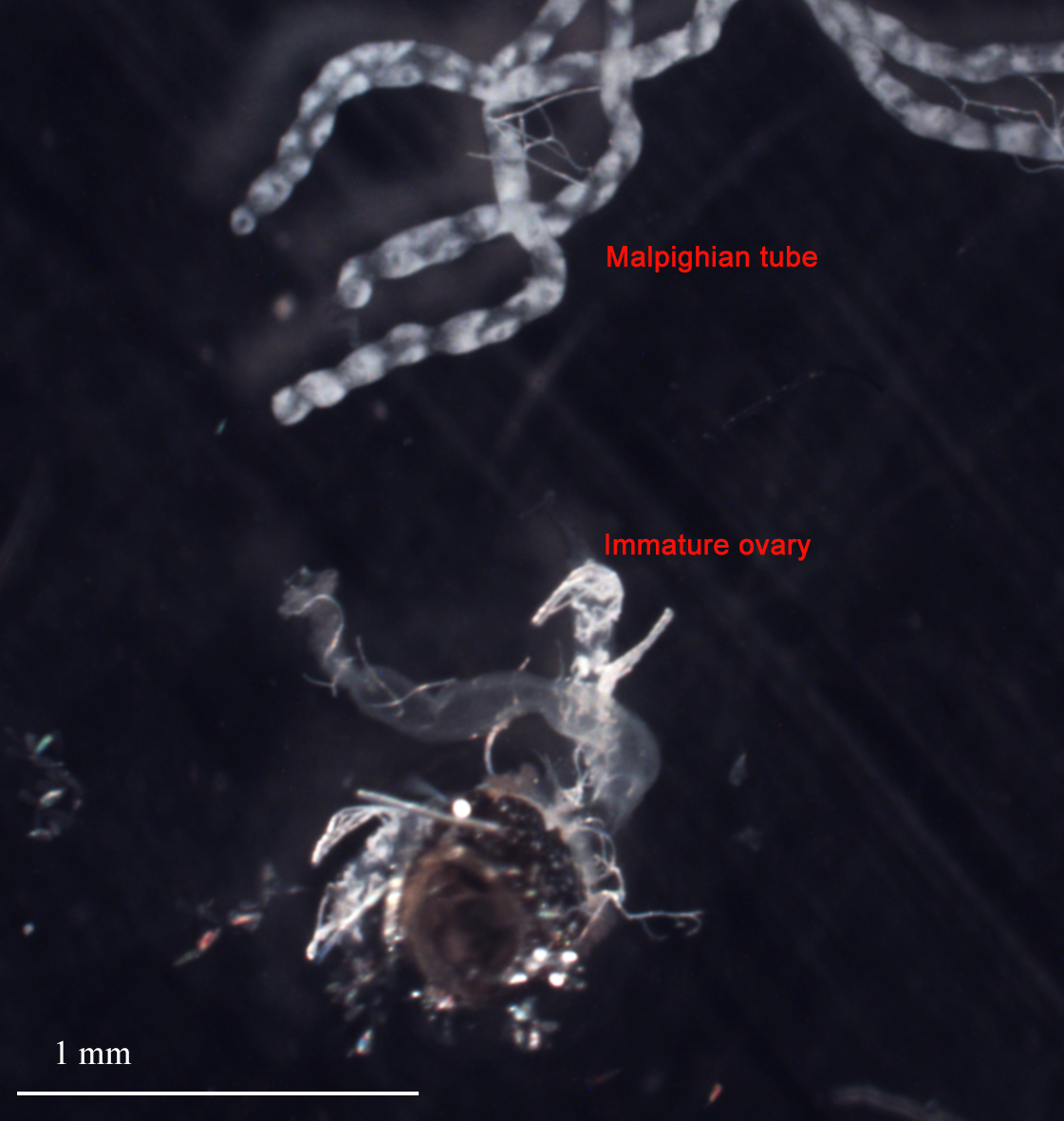

Supplement: S1 Fig — (DOCX) [file pntd.0010913.s007.docx]
