# Supplementary material for: Wolbachia inhibits ovarian formation and increases blood feeding rate in female Aedes aegypti
Source: PLoS Negl Trop Dis. 2022 Nov 11;16(11):e0010913. doi: 10.1371/journal.pntd.0010913 (PMC9683608; doi:10.1371/journal.pntd.0010913)
Supplement: S2 Fig — (DOCX) [file pntd.0010913.s008.docx]

**S2 Fig.** Box plots of relative *Wolbachia* density of fertile and infertile *w*AlbB-infected females after females were separated into fertile and infertile groups (one week after blood meal).

Densities are based on two consistent real-time PCR replicates of 16 females from each group. Fertile females had higher relative densities of *Wolbachia* (ANOVA: F_1,30_ = 8.632, P = 0.006, fertile: mean ± se = 5.15 ± 0.906; infertile: mean ± se = 2.290 ± 0.673).
