# Supplementary figures and images for: Wolbachia inhibits ovarian formation and increases blood feeding rate in female Aedes aegypti
Source: PLoS Negl Trop Dis. 2022 Nov 11;16(11):e0010913. doi: 10.1371/journal.pntd.0010913 (PMC9683608; doi:10.1371/journal.pntd.0010913)

**S3 Fig.** Original pictures for Fig 1.


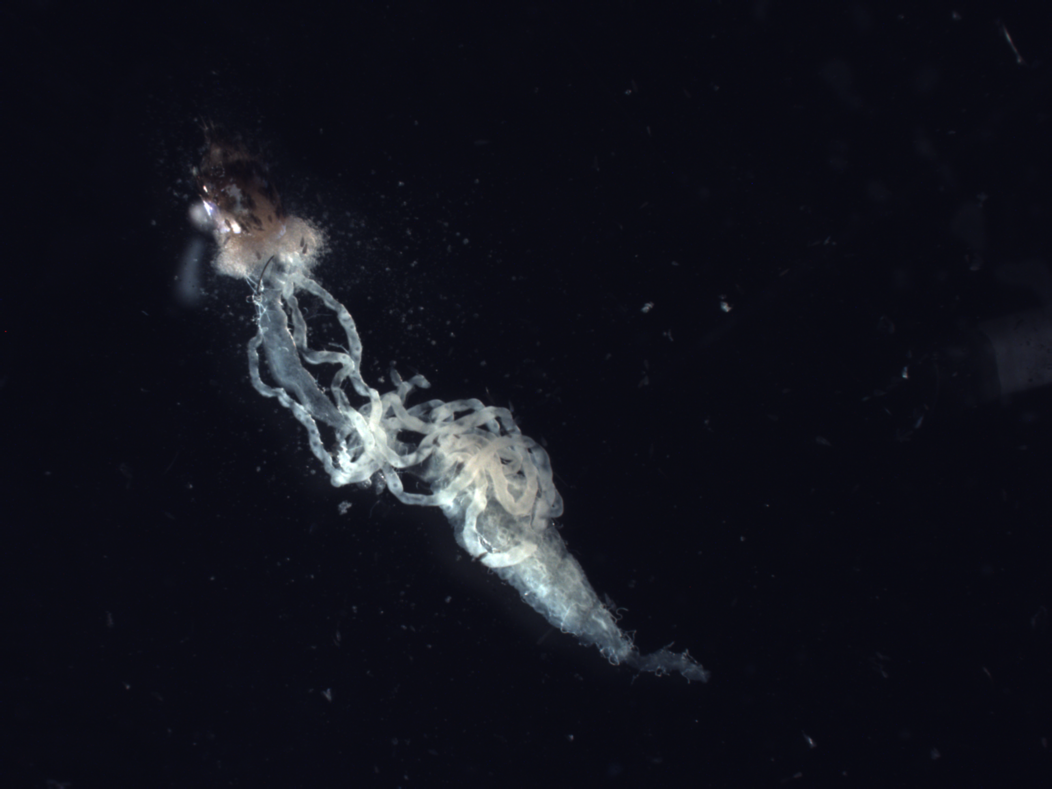


Original picture of Fig 1A.


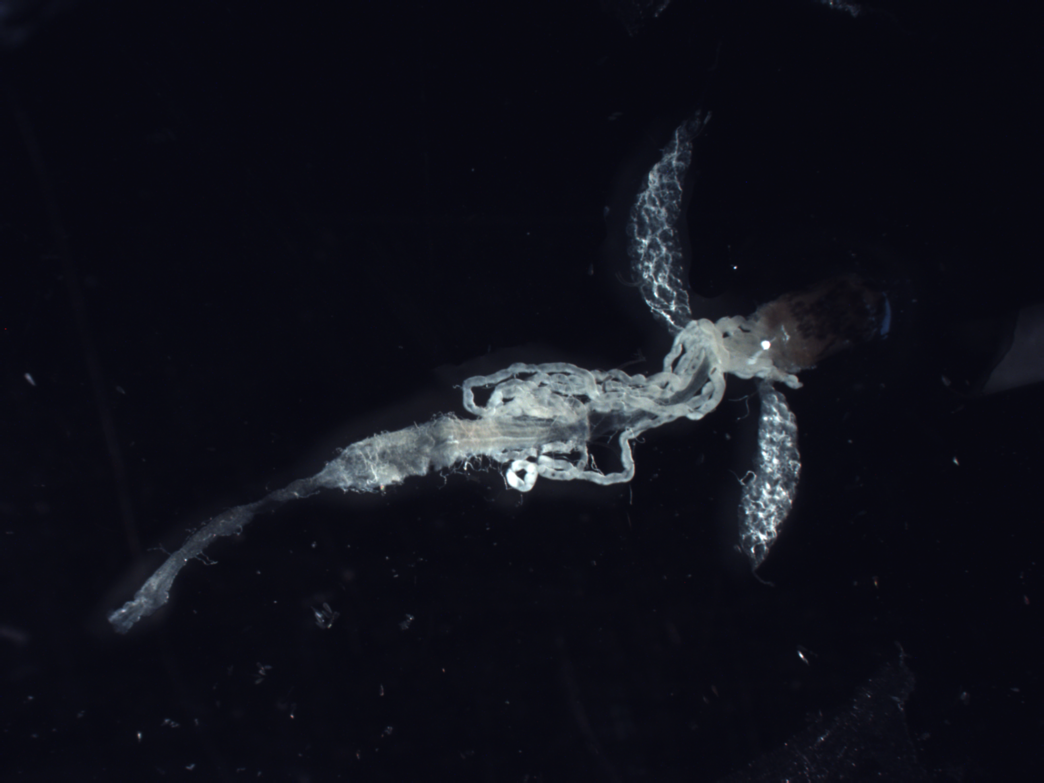


Original picture of Fig 1B.

Supplement: S3 Fig — (DOCX) [file pntd.0010913.s009.docx]
